# Role of prior intratympanic gentamicin and corticosteroids therapy on speech understanding in patients with Menière's disease after cochlear implantation

**DOI:** 10.1007/s00405-024-08449-8

**Published:** 2024-02-02

**Authors:** Kruthika Thangavelu, Frederic Gillhausen, Rainer M. Weiß, Jochen Mueller-Mazzotta, Boris A. Stuck, Katrin Reimann

**Affiliations:** Department of Otorhinolaryngology, Head and Neck Surgery, University Hospital Marburg, Philipps-Universität Marburg, Baldingerstrasse, 35043 Marburg, Germany

**Keywords:** Meniere’s disease, Intratympanic corticosteroids, Intratympanic gentamicin, Cochlear implantation, Post-operative hearing

## Abstract

**Aim:**

Intratympanic injection of corticosteroids (ITC) and gentamicin therapy (ITG) are widely used treatments for vertigo in Meniere’s disease (MD). Even though studies show good results after cochlea implantation (CI) in MD patients when compared to non-MD groups, there is no indication on the effect of ITC and ITG prior to CI on hearing after CI. This study compares the post-operative hearing of CI patients with and without MD and patients who have received ITG or ITC prior to CI.

**Methods:**

In a retrospective case control study, adult patients with MD who received CI from 2002 till 2021 were compared to a matched control group of CI patients without MD. Patients with prior ITC/ITG were extracted from MD group. Pre-operative audiological results were measured and trends across post-operative monosyllabic word recognition score at 65 decibels (WRS65CI) at switch-on, 3–6 months, 1 year and last yearly value were analyzed across all groups.

**Results:**

28 MD ears were compared with 33 control ears. From MD ears 9 had received ITG and 6 ITC prior to CI. WRS65CI increased significantly with time within MD and control groups, but no difference in WRS65CI was found between these 2 groups. ITG ears showed fluctuating WRS65CI after CI with no change across time, while ITC ears showed significant increase in trend of WRS65CI values across time.

**Conclusion:**

MD and non-MD patients showed comparable hearing results after CI. Prior ITC might positively influence hearing preservation after CI in MD patients whereas ITG group showed fluctuating hearing.

## Introduction

Meniere’s disease is the disease of the inner ear which is usually characterized by episodic vertigo, fluctuating sensorineural hearing loss, tinnitus, and fullness on the affected side of the ear [26]. The diagnosis of the disease is challenging due to the lack of a gold-standard diagnostic test. The agreed upon classification based on clinical symptoms helps in diagnosing ‘definite’ or ‘probable’ disease [24] Even though the disease prevalence is estimated to be 10–150 per 100,000 the difficulties in diagnosis makes it difficult to ascertain the true level of prevalence [4, 16]. It has been observed that the disease is more common in the fourth and fifth decades of life, with a higher incidence in females and Caucasians.

The diagnosis of definite MD is made with the following criteria: (1) two or more spontaneous episodes of vertigo lasting between 20 min and 12 h, (2) documented low- to mid-frequency SNHL during or after an episode of vertigo, (3) fluctuating symptoms in the affected ear, and (4) the aforementioned symptoms are not better accounted for by another vestibular disorder [26].

The first line management for symptomatic patients is usually conservative involving dietary changes such as reducing salt intake, caffeine, alcohol, and nicotine; and oral treatment with Betahistine or diuretics or a combination all of them with diuretics showing the least evidence [26]. Second line of treatment with intratympanic injection of corticosteroids (ITC) is considered when patients do not respond to medical management [34]. The third line treatment involving invasive endolymphatic sac surgery even though still widely performed is controversial with little evidence [23, 36]. The fourth line treatment is the partial or total medical ablative treatment with intratympanic gentamicin (ITG) usually reserved for patients who already suffer from considerable hearing loss [1]. The fifth line treatment is the total surgical ablation by surgical labyrinthectomy or vestibular neurectomy. The combination of surgical labyrinthectomy with cochlear implantation is advised in order to rehabilitate the hearing of the patients [42].

Regardless of the treatments offered, a large portion of the disease population will progress to profound hearing loss, resulting in functional deafness in one or both ears [19]. This makes hearing rehabilitation an important point to consider in these patients, with cochlear implantation gaining momentum in recent years as a viable treatment option.

On one hand, studies have shown varying degrees of post-operative success in hearing rehabilitation in these patients after cochlear implantation [25, 31, 38] and on the other hand there is a lack of long-term follow-up [8]. There is a specific lack of studies involving the effect of ITC or the ablative effect of ITG on the hearing rehabilitation with cochlear implantation. This is especially important considering that ITG and ITC are probably the most effective non-surgical treatments to treat vertigo in MD [47].

In this scenario before offering treatments with gentamicin and/or ITC, it is important to ascertain their short and long-term effects on hearing rehabilitation with CI since it is usually the last chance of hearing rehabilitation available to the patients. The aim of this study is to compare the post-operative speech understanding of patients who received CI with and without MD. It also aims to analyze the influence of ITG and ITC on the post-operative speech understanding of MD patients.

## Methods

### Study design and population

This is a retrospective case control study involving chart review of all patients (adults of at least 18 years of age) who were diagnosed with MD and received CI at a tertiary care cochlear implantation center from January 2002 till December 2021. The diagnosis of definite MD and/or probable MD was done based on the criteria outlined by the current international guidelines [41]. The symptoms available at the time of diagnosis were reviewed again using patient documents and only patients with definite and/or probable MD were included in the study. This data also included the general demographics of the patients, the type of prior treatments for MD such as oral Betahistine, diuretics, ITC, ITG, operative intervention including endolymphatic sac decompression, labyrinthectomy and vestibular nerve section. The age at CI and electrode type used was also extracted from the patient documents. A matched cohort of CI patients without MD was extracted from our databank as controls based on age, pre-operative hearing threshold levels and duration of hearing loss.

### Audiological data

The pre-operative pure tone audiometry and speech understanding were recorded. The bone conduction pure tone audiometry was measured at 500, 1000, 2000 and 4000 Hz and the average which was termed as pure tone average (PTA4) was calculated. The Freiburg monosyllabic speech test commonly used in German speaking countries was performed at various decibels prior to implantation with and without hearing aids before implantation. The Freiburg monosyllabic test is conducted with headphones within the standardized speech audiogram and in free field with/without hearing aids or with CI. This provides information about speech intelligibility at conversation levels and at the maximum possible frequency close to discomfort level.

At the center where the study was conducted, the unaided maximum recognition score (WRSmax) for phonemically balanced monosyllabic words is routinely measured for all patients who are in need of hearing rehabilitation. This is measured at near discomfort level using air-conduction headphones, which is usually at 110 decibels. Following WRSmax, the score for recognition of phonemically balanced monosyllabic words at conversation level of 65 dB with best possible hearing aids is measured (WRS65HA). WRS65HA is usually measured in free field in a non-echoic booth at 65 dB. The contralateral ear is always masked appropriately with wideband noise also presented using headphones. For the purpose of this study these two values (WRSmax and WRS65HA) were extracted for all patients.

Similarly, the post-operative hearing after CI is measured for all patients at the center using the Freiburg phonemically balanced monosyllabic words test at conversation level of 65 dB (WRS65CI). The contralateral ear is always masked appropriately with wideband noise also presented using headphones. The WRS65CI is measured sequentially at switch-on which is usually at 3 weeks after implantation (FU1), then 3–6 months after implantation (FU2), one year after implantation (FU3) and then was measured yearly. All of the above mentioned WRS65CI values were extracted for this study and the last available yearly measurement extracted was termed as FU4.

### Statistical analysis

Categorical variables were analyzed using a Fisher exact or Chi-square test. Continuous variables were tested for normal distribution using Shapiro Wilk test and were analyzed using a Student t test. Nonparametric data were analyzed using Mann–Whitney U tests. Preoperative speech and hearing data variables across various groups were compared to postoperative cochlear implant speech and hearing data using a Student t test or Mann–Whitney U tests. Further statistical analyses were conducted to determine the effect of categorical and continuous variables including patient’s age and prior intervention on both speech and hearing postoperative outcomes. A repeated measures ANOVA was used to test the effects of prior treatments on the post CI hearing values followed by a non-parametric Mann–Kendall trend test to detect monotonic trends across the post CI hearing levels. Group differences were considered significant if p value was less than 0.05. All analyses were performed using Stata 14.0 (StataCorp. 2014. Stata Statistical Software: Release14. College Station, TX: StataCorp LP).

## Results

### Patient characteristics

In total 579 patients received CI at the tertiary care cochlear implantation center from January 2002 till December 2021. 26 patients were found to be diagnosed with definite MD unilaterally and 1 patient with definite MD on one side and probable MD on the contralateral side and thus 28 ears with MD were included in this study. Another 26 patients were identified from the same CI databank who did not have MD and were matched with the MD patients based on age, level of hearing loss and duration of deafness. 7 patients from the non-MD group received bilateral sequential CI and thus 33 implanted ears were included in the control group. The average age of MD patients at the time of CI surgery was 55.7 ± 15.7 years with 12 female patients, the median age being 58 years. The average age of the control group at the time of CI surgery was 54.1 ± 17.4 years, with a median age of 56 years, showing no statistical difference between the two groups (Table 1). All of the patients included were found to have had no vertigo episodes with-in the 6 months prior to CI.

**Table 1:** Patient descriptive

	Total MD *N* = 28*	ITG *N* = 9	ITC *N* = 6	Control group *N* = 33^+^
Age (years) mean ± SD median	55.7 ± 15.7	50.8 ± 11	48.2 ± 19.4	54.1 ± 17.4
No. of female patients	12	11	2	12
Right side	18	5	4	18
Type of CI device				
Med El				
Flex 28	5			9
Cochlear				
Contour Advance	6			4
Slim Straight	15			19
AB SlimJ	2			0
Oticon	0			1

*MD* Meniere’s Disease, *ITG* intra-tympanic treatment with gentamicin, *ITC* intra-tympanic treatment with corticosteroids, SD = standard deviation

^*^27 patients were included among which one patient received bilateral CI with bilateral Meniere’s disease, therefore 28 ears were in total analyzed

^ +^ 26 patients were included among which 7 patients received bilateral CI, therefore 33 ears were in total analyzed

All the 27 MD patients were documented to have been advised a conservative treatment initially with dietary changes and decreased salt intake. Betahistine was found to be prescribed in varying doses to 14 (53.8%) patients with or without diuretics, but the information was vague with no specific dosage or period of the treatment. In case of second line invasive interventions, 6 ears (21.4%) were documented to have received prior treatment with intratympanic corticosteroids. The third line operative endolymphatic sac decompression was performed in 9 (32.1%) ears. Nine (32.1%) ears underwent the fourth line medical ablative procedure with gentamycin before receiving CI. The fifth line operative vestibular nerve resection or labyrinthectomy was found to be done in none of the patients.

The patients were noted to have received multiple treatments, for example Betahistine combined with intratympanic corticosteroids and then followed by endolymphatic sac decompression, or conservative treatments followed by intratympanic gentamicin treatment. But ITG and ITC showed minimal overlap, with only one patient with unilateral MD having received both ITC and ITG. Four patients (14.8%) had received only the conservative first line management without any sort of invasive intervention. Majority were implanted with electrodes from Cochlear® (72.1%, *n* = 44; slim straight 34, contour advance 10), followed by Flex 28 electrodes from Med-EL® (23%, *n* = 14) and SlimJ from Advanced Bionics® (3.3%, *n* = 2) (Table 1).

### Audiological results before cochlear implantation

The pre-operative audiogram results were found to be normally distributed across all groups. The mean PTA4 prior to CI in the MD group was better at 87.4 ± 25.2 dB (median: 79.8) compared to 95.3 ± 24 dB (median: 93.8) in the control group, with no statistically significant difference in the two-sample *t* test. On the other hand, although there was no statistically significant difference, the WRSmax and WRS65HA were better in the control group than the MD group (Table 2). The ITG and ITC groups separately were found to have very similar pre CI PTA4, WRSmax and WRS65HA values to the MD as a whole as shown in Table 2.

**Table 2:** Hearing across various groups before hearing rehabilitation with cochlea implantation

	Total MD *N* = 28 (mean ± SD) median	MD without ITG/ITC *N* = 14 (mean ± SD) median	ITG Group *N* = 9 (mean ± SD) median	ITC Group *N* = 6 (mean ± SD) median	Control Group *N* = 33 (mean ± SD) median
PTA4 (dB)	87.4 ± 25 79.75	92.2 ± 29 87	89.9 ± 23 81.5	74.7 ± 18.2 71.5	95.3 ± 24 93.75
WRSmax (%)	19.1 ± 27 10	14.7 ± 26 5	22.5 ± 35 10	24.2 ± 19 22.5	14 ± 21 5
WRS65HA (%)	4.6 ± 8.9 0	4.6 ± 11.3 0	5.6 ± 6.8 5	3.3 ± 6.1 0	3.5 ± 8.8 0

*MD* Meniere’s Disease, *ITG* intra-tympanic treatment with gentamicin, *ITC* intra-tympanic treatment with corticosteroids, *SD* standard deviation, *PTA4* pure tone average, *WRSmax* unaided maximum recognition score, *WRS65HA* score for recognition of phonemically balanced monosyllabic words at conversation level of 65 dB with best possible hearing aids

### Monosyllabic Word Recognition score after cochlear implantation

The average follow-up time for the MD after CI was 5.1 ± 4.2 years and for control group was 5.1 ± 4.9 years. When comparing the MD patients with the control group, the control group performed slightly better than the MD ears across all the four follow-up time points but without any statistically significant difference (Table 3). The repeated measures ANOVA showed statistically significant changes in the WRS65CI across time within the groups (F value 32.4, p value 0.00), and there was no significant difference in WRS65CI between groups.

**Table 3:** Monosyllabic Word Recognition score in percentage after cochlear implantation at 65 dB (WRS65CI) across 4 follow-up time points among Meniere group (MD) and control group

	WRS65CI at FU1 (%)	WRS65CI at FU2 (%)	WRS65CI at FU3 (%)	WRS65CI at FU4 (%)
MD group				
Mean ± SD	34.2 ± 25.6	52.9 ± 25.1	56.4 ± 26.7	61.7 ± 27.7
Median	30	55	65	65
Control group				
Mean ± SD	26.0 ± 26.4	57.4 ± 28.3	64.2 ± 25.2	65.2 ± 29.2
Median	20	60	70	75

*FU1* at switch-on, *FU2* at 3-6 months after implantation, *FU3* 1 year after implantation, *FU4* last yearly measurement available

An overall statistically significant difference of 43% was found between mean pre- and post-operative speech recognition after 1 year (95% CI 27.0–58.2, *p* = 0.00) in the MD group.

The MD group was further classified into ears having received prior ITC or prior ITG or neither. The mean WRS65CI for the MD group without prior ITC or ITG across the 4 follow-up time points were 30.4 ± 23.7%, 42.9 ± 26.3%, 56.9 ± 29.2% and 55.4 ± 31.0% respectively. Whereas the control group showed following WRS65CI values: 26.0 ± 26.4%, 57.4 ± 28.3%, 64.2 ± 25.2% and 65.2 ± 29.2%. This shows that MD ears without prior ITC or ITG performing worse over time compared to the control group even though the individual mean values did not show any statistically significant difference.

**Fig. 1:**
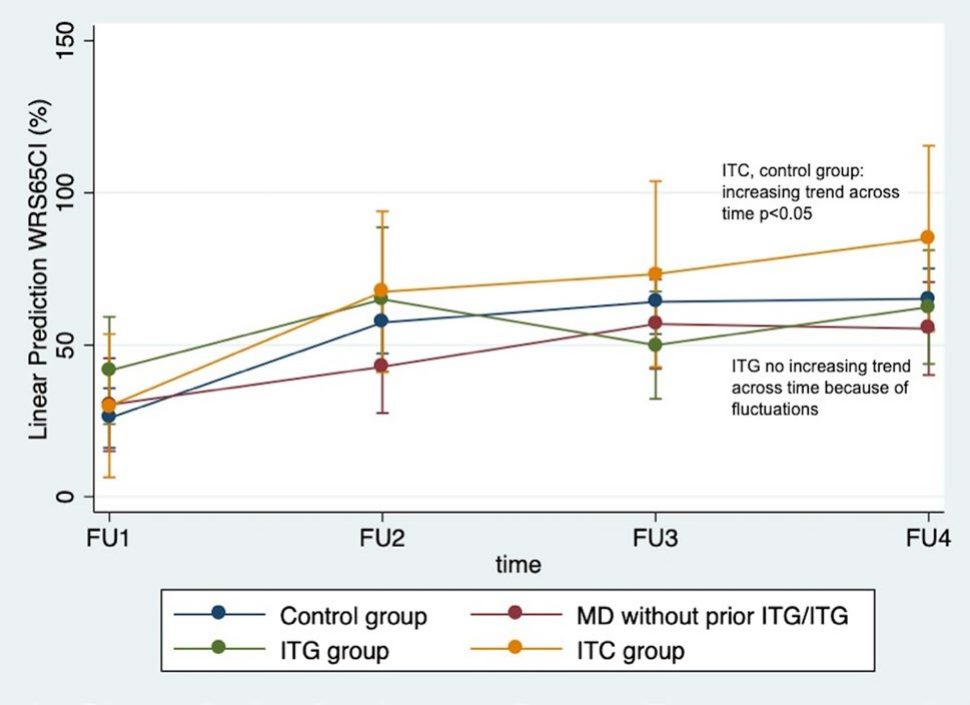
Adjusted predictions of WRS65 CI for prior treatment with 95% confidence interval showing trends across time. *WRS65CI* Monosyllabic Word Recognition score in percentage after cochlear implantation at 65 dB, *FU1* at switch-on, *FU2* at 3-6 months after implantation, *FU3* 1 year after implantation, *FU4* last yearly measurement available

The ITG ears showed fluctuating results after CI over the follow-up time periods as follows: 41.7 ± 29.4%, 65 ± 20.3%, 50 ± 25.1% and 62.5 ± 23.5%. The ITC group performed better than all other groups over time with statistically significant difference between the first and second follow-up mean WRS65CI values in two sample t test as follows: 30 ± 25.5%, 67.5 ± 13.2%, 73.3 ± 17.6% and 85 ± 13.2% (Fig. 1). A two-way repeated measures ANOVA showed that time as a within group factor had a statistically significant effect on WRS65CI (*F* value 20.8, p value 0.00), but the prior treatments (ITG, ITC, or no prior treatment) as the between groups factor showed no significant effect on WRS65CI values (*F* value 0.37, *p* value 0.77).

The median WRS65CI values from the ITC and control group showed a statistically significantly increasing trend across time in the Mann Kendall test for trend overview (Kendall’s score 6, *p* value 0.044) (Fig. 1). The MD group without prior ITC or ITG also showed an increasing trend but was not statistically significant (Kendall’s score 5, *p* value 0.148), whereas the ITG group showed no such trend at all (Kendall’s score 0, *p* value 1.000) (Fig. 1). After categorizing age across various points, age was not found to have any significant effect on the outcome WRS65CI values.

## Discussion

Hearing rehabilitation with CI is in the meantime routinely used to treat hearing loss in patients with MD. Although initial reports showed varied results compared to standard CI patients [25, 31, 38], larger studies including systematic reviews show comparable results in speech understanding to non-MD patients [2, 44]. These results could be reproduced in our study especially in terms of post-operative speech understanding.

When comparing with recent studies in detail, Prenzler et al. in 2017 report that among 27 MD patients implanted with CI, MD patients showed significantly better results in speech understanding compared to controls at the first fitting and at 1-year refitting, this effect could not be seen anymore [35]. Although not statistically significant, this result was similar to our study wherein the MD patients showed better speech understanding at initial fitting than the controls, but controls performed better with time. The study also showed that pre-operative PTA4 was better in MD patients compared to controls, which was similar to our study. Despite this, these patients did not benefit from best hearing aids (WRS65HA). This has been accounted to fluctuating hearing loss [28, 30, 43]. In addition, this also hints at a possible acoustic degradation over time in MD patients after CI. In fact, Masood et al. in 2020 reported that even though 27% of the MD patients achieved short-term functional hearing preservation, the long-term outcomes were less favorable. They also suggest that there can be degradation in acoustic hearing over time [27].

Another study by Chien et al. in 2022 report that among 29 Meniere patients who received CI, an overall statistically significant difference of 56% was found between mean pre- and post-operative speech recognition after 1 year, which is similar to our study [5]. The study also reports increasing mean post-operative scores at 1 month, 3 months, 6 months, 1 year, and after 1 year at 37.1%, 46.1%, 54.1%, 59.1%, and 66.8% respectively which are similar to our study although a repeated measures ANOVA analysis is missing in this study. However, the higher mean post-operative hearing improvement in patients aged less than 70 years when compared to older MD patients reported by Chen et al. was not seen in our study. In contrast our study showed that age did not have any influence on the post-operative hearing outcomes despite similar study population.

Our study shows varying post-operative speech understanding results after CI when comparing MD patients who received ITG or ITC with MD patients who received neither. The MD ears without prior ITC or ITG performed slightly worse over time compared to the control group but could be said to be comparable to controls since there was no statistical difference between individual means across time points. But the surprising results were from the MD patients treated with ITG or ITC prior to CI. Our study suggests fluctuating speech understanding in patients treated with ITG whereas the ITC patients show very promising speech understanding with dramatic improvement over time.

ITG even though reserved for MD patients with refractory vertigo and with considerable hearing loss, is still more widely used than ITC because of two reasons: ‘chemical labyrinthectomy’ with aminoglycosides is a well-known concept since introduced in 1948 by fowler [11] whereas ITC is a recent treatment method introduced in 2007 [3]. The second reason is that the evidence of disease control for ITC in MD patients is still rather questionable as shown in the Cochrane and systematic reviews [9, 45, 47] with very few studies showing control of disease symptoms [18, 21]. What needs to be considered here is that when ITG might be better at controlling vertigo, ITC has been shown to be better at hearing preservation but not effective in controlling vertigo. This is supported by the pathogenesis of the aminoglycosides, especially gentamicin, which is more sensitive to vestibular hair cells than cochlear hair cells [37, 39]. This deferential toxicity helps reducing vertigo attacks. The steroids on the other hand have shown benefits since immune-mediated response might also be a factor that causes the onset of MD [7, 10, 13]. Corticosteroids furthermore have been found to influence the ionic hemostasis regulation through modifications in the potassium transport consequently reducing the damage to intracochlear barrier [20]. In addition, steroids have been found to play a protective role by the water balance in the inner ear by aquaporins resulting in a possible hearing preservation [12]. Not to be forgotten at this point is that even though cochlear implants bypass the membranous labyrinth they are said to rely on the spiral ganglion for functionality [40]. Steroids may also help in reducing the loss of spiral ganglion neurons helping in hearing preservation [46].

The phenomenon of fluctuating post-operative hearing in MD patients with prior ITG treatment shown in our study has already been reported by case reports [17,29]. The underlying mechanism for fluctuation in hearing in MD patients is still not clear. It has been hypothesized that endolymphatic hydrops might alter the electrode position or disturb the electrode- neural interaction by changing the endolymph fluid potential. This fluctuation occurring only in ITG group and not in ITC group in our study is still harder to explain. We can only speculate that the ITC group as suggested by the prior pathogenesis plays a hearing protective role, which the ITG group lacks [14, 22, 32]. There has also been increased discussion in the last years suggesting that ITG might be more effective in controlling vertigo episodes than ITC with negligible hearing loss when used in low doses [6, 33]. Unfortunately, no current consensus exists on clinical guidelines for the use of gentamicin in terms of dose and duration. Thus, even though there is enough evidence for intratympanic drug treatment for Menière’s disease using ITG and ITC, proper counseling is needed in these patients especially about expectations of post-operative speech understanding when considering hearing rehabilitation with CI.

The role of active disease in MD should also be considered when discussing fluctuations in hearing. MD usually ‘burns out’ in the later phase with patients showing a hearing threshold of up to 60 dB or more pan-tonally resulting in a functionally deaf ear with much decreased speech understanding following an irreversible loss of the hair cells [15]. This is also the stage when the patients receive CI resulting in a lesser possibility of fluctuation in hearing. Notably though, the ‘attacks’ are found to persist, wherein the patients still report the “feeling of fullness” in the ear but without hearing loss or typical vertigo episodes. We have tried to clinically rule out active disease being a cause of the fluctuation by including only patients without a vertigo episode with-in 6 months prior to CI. Since ITG is said to mimic the ‘burned out’ phase resulting in a similar irreversible loss of the hair cells, the fluctuation seen in the ITG group in our study should be of concern needing further studies.

The small study population with heterogeneity is a limitation of this study. However MD being a rare disease, achieving a bigger study population in a single center has always been problematic. This necessitates more studies preferably multicentric with bigger study groups especially for ITG/ITC groups. The lack of the inclusion of vertigo control is another important limitation of the study since vertigo control is the main goal of the ITC and ITG treatments. The influence of different surgical techniques and electrodes on outcomes after CI should also be mentioned as possible confounding factors. Fortunately all the CIs in our center were done by specialized CI surgeons using similar techniques eliminating this factor. The influence of different electrodes used though could not be analyzed because of the small study population. Despite these limitations as far as we know this is the first study on the role of ITG and ITC on speech understanding in patients with Menière's disease after cochlear implantation.
